# Extracellular Vesicles Derived from Primary Adipose Stromal Cells Induce Elastin and Collagen Deposition by Smooth Muscle Cells within 3D Fibrin Gel Culture

**DOI:** 10.3390/bioengineering8050051

**Published:** 2021-04-27

**Authors:** Eoghan M. Cunnane, Aneesh K. Ramaswamy, Katherine L. Lorentz, David A. Vorp, Justin S. Weinbaum

**Affiliations:** 1 Department of Bioengineering, University of Pittsburgh, Pittsburgh, PA 15260, USA; eoc2@pitt.edu (E.M.C.); akr40@pitt.edu (A.K.R.); kll68@pitt.edu (K.L.L.); vorp@pitt.edu (D.A.V.); 2 Tissue Engineering Research Group, Department of Anatomy and Regenerative Medicine, Royal College of Surgeons in Ireland, Dublin, Ireland; 3 McGowan Institute for Regenerative Medicine, University of Pittsburgh, Pittsburgh, PA 15260, USA; 4 Department of Surgery, University of Pittsburgh, Pittsburgh, PA 15260, USA; 5 Department of Chemical and Petroleum Engineering, University of Pittsburgh, Pittsburgh, PA 15260, USA; 6 Department of Cardiothoracic Surgery, University of Pittsburgh, Pittsburgh, PA 15260, USA; 7 Department of Pathology, University of Pittsburgh, Pittsburgh, PA 15260, USA

**Keywords:** aneurysm, fibers, modulus, vasculature, regenerative medicine

## Abstract

Macromolecular components of the vascular extracellular matrix (ECM), particularly elastic fibers and collagen fibers, are critical for the proper physiological function of arteries. When the unique biomechanical combination of these fibers is disrupted, or in the ultimate extreme where fibers are completely lost, arterial disease can emerge. Bioengineers in the realms of vascular tissue engineering and regenerative medicine must therefore ideally consider how to create tissue engineered vascular grafts containing the right balance of these fibers and how to develop regenerative treatments for situations such as an aneurysm where fibers have been lost. Previous work has demonstrated that the primary cells responsible for vascular ECM production during development, arterial smooth muscle cells (SMCs), can be induced to make new elastic fibers when exposed to secreted factors from adipose-derived stromal cells. To further dissect how this signal is transmitted, in this study, the factors were partitioned into extracellular vesicle (EV)-rich and EV-depleted fractions as well as unseparated controls. EVs were validated using electron microscopy, dynamic light scattering, and protein quantification before testing for biological effects on SMCs. In 2D culture, EVs promoted SMC proliferation and migration. After 30 days of 3D fibrin construct culture, EVs promoted SMC transcription of the elastic microfibril gene FBN1 as well as SMC deposition of insoluble elastin and collagen. Uniaxial biomechanical properties of strand fibrin constructs were no different after 30 days of EV treatment versus controls. In summary, it is apparent that some of the positive effects of adipose-derived stromal cells on SMC elastogenesis are mediated by EVs, indicating a potential use for these EVs in a regenerative therapy to restore the biomechanical function of vascular ECM in arterial disease.

## 1. Introduction

Cardiovascular disease (CVD) is the primary cause of death globally, outside of the current COVID-19 pandemic, and encompasses disorders of the heart and blood vessels [1]. Affected vessels can exhibit aneurysm or blockage and often require revascularization through minimally invasive endovascular repair or open bypass surgery [2,3]. Despite continuous advancements in the field, limitations to CVD treatment persist. For example, non-surgical options for patients with small abdominal aortic aneurysm (AAA) (diameter below the “surgical” threshold) are limited to semi-annual surveillance, with current pharmacological agents proving ineffective and AAA rupture occurring in nearly a quarter of patients in this cohort [4,5,6]. Additionally, the autologous and synthetic grafts utilized for bypassing small diameter arteries (<6 mm) have critical flaws; autologous grafts are often unavailable while both graft types have high short term failure rates due to thrombosis and hyperplasia [7,8,9,10]. Further reading on the development of tissue engineered/regenerative medicine solutions for these needs has been highlighted in reviews [11,12]. In either case, it is paramount to develop methods of inducing the synthesis of the key extracellular matrix (ECM) structural proteins, elastin and collagen, in target vascular cells and tissues.

Vascular elastin is formed by mature smooth muscle cells (SMCs) just prior to birth and during the early postnatal period. The expression of tropoelastin (core protein) and elastin organizational matricellular proteins (members of the fibrillin, fibulin, lysyl oxidase, and latent transforming growth factor β binding protein, or LTBP families) is subsequently downregulated during adulthood [13,14]. At the biomechanical level, elastin is responsible for the passive recoil of vascular tissue during pulsatile loading, and elastin fiber degradation is a key feature of aneurysm dilation. Fibrillar collagens are synthesized by vascular SMCs and adventitial fibroblasts, then post-translationally modified by proteins of the prolyl hydroxylase and LOX families before macromolecular assembly into a triple helical fiber as reviewed in [11]. Biomechanically, fibrillar collagens provide vascular tissue with the mechanical strength necessary to prevent rupture during supraphysiological loading. The presence of properly assembled collagen fibers is essential to the formation of viable de novo tissue intended for use as a bypass graft. The development of a method to induce elastin and collagen production in SMCs is therefore a challenging, yet highly desirable, target for vascular tissue engineering and regenerative medicine.

We have previously demonstrated that delivering the secretome of adipose stromal cells (ASCs) to vascular SMCs in 3D gel culture increases the deposition of both elastin and collagen [15]. Others have shown a potent effect of mesenchymal stem cells [16] and their extracellular vesicles (EVs) [17] on reducing aneurysm growth, furthering the premise of this study. Herein, we further elucidate the fraction of the ASC secretome that induces ECM synthesis in SMCs by treating the cells with EVs derived from ASCs. We hypothesize that EVs will have a potent ECM protein synthesis effect on SMCs as EVs derived from stromal cells are specifically enriched in cargo for extracellular matrix remodeling [18,19,20]. We utilized our fibrin-based 3D SMC culture platform to test our hypothesis and demonstrated that administering ASC-derived EVs to SMCs increases the deposition of collagen and elastin, thus highlighting that EVs can be employed as a cell-free therapeutic tool to assist in developing novel treatments aimed at arresting aneurysm growth and fabricating vascular bypass graft material.

## 2. Materials and Methods

### 2.1. ASC Culture Conditions and Conditioned Media Collection

Primary ASCs were obtained from de-identified waste human adipose tissue collected during body sculpting surgeries at UPMC Presbyterian Hospital. All patients were non-smoking, non-diabetic, and <45 years old. Approximately 100 mL of human adipose tissue was minced with scissors and digested in collagenase (1 mg/mL) and bovine serum albumin (BSA, 35 mg/mL, protease free heat shock, Equitech-Bio Inc. #BAH65, Kerrville, TX, USA) followed by filtration and heating within a 37 °C shaker bath for one hour [21,22]. The suspension was homogenized via repeated inversions and passed through a 0.5 mm gauze (10 × 10 cm, Fisher #22-415-469). The tubes were centrifuged at 250× *g* at 4 °C for 10 min, the supernatant was discarded, and the pellet was resuspended in 10 mL of red blood cell lysis buffer (ACK Lysing Buffer, ThermoFisher Scientific #A10492-01, Waltham, MA, USA). The suspension was passed through a sieve to remove any remaining particles (500 µm, pluriSelect #43-50500-01) and centrifuged again under the same conditions. The supernatant was removed using a vacuum pipet tip and the pellet was resuspended in ASC non-conditioned media (nCM), consisting of 33% Dulbecco’s Modified Eagles Medium (High Glucose DMEM, Gibco #12100046), 33% DMEM/F12 Medium (HEPES, Gibco #12400024), 7.5% fetal bovine serum (FBS, Premium Select, Atlanta Biologics #S11550), 0.75% fungizone (Lonza BioWhittaker Antiobiotics #BW17836E), 0.75% penicillin/streptomycin (10,000 U/mL, ThermoFisher Scientific #15140122), 0.075 μM dexamethazone (Sigma-Aldrich #D4902, St. Louis, MI, USA), and 25% Preadipocyte Growth Medium (PromoCell #C-39425, Heidelberg, Germany). The FBS was first depleted of EVs via ultracentrifugation at 120,000× *g* for 18 h at 4 °C. Then, 6 million cells were seeded into two 5-layer T-175 tower flasks (equivalent to approximately 3500 cells/cm^2^) and cultured for 18 h in 75 mL of nCM. The cells were rinsed in phosphate buffered saline (PBS, pH 7.4, Gibco #10010023) and 110 mL of fresh nCM was added to each tower. The media was collected after 48 h and referred to as ASC conditioned media (CM).

### 2.2. EV Isolation

The CM was immediately subjected to centrifugation at 250× *g* (20 min at 4 °C) to remove cells followed by 2500× *g* (20 min at 4 °C) to remove cell debris. The CM was then filtered through 0.22 μm filters (Express PES Membrane, Millex #SLGP033RS, Merck KGaA, Darmstadt, Germany) to remove apoptotic bodies and 34 mL of the processed CM was added per ultracentrifuge tube (Polypropylene 25 × 89 mm, 6 tubes total, Beckman Coulter # 326823). The tubes were inserted in metal cases, hung on a swinging bucket rotor (SW28.1, 117.1 g cases, Beckman Coulter, Brea, CA, USA), and centrifuged at 100,000× *g* (70 min at 4 °C) to pellet the EVs using an ultracentrifuge (L8-70M, Beckman Coulter, Brea, CA, USA). The supernatant was removed and stored as EV depleted conditioned media (dCM). The tubes were overturned on a sterile drape to drain remaining supernatant and residual droplets were removed with a vacuum tip. The pelleted EVs were re-suspended in 100 μL of 4 °C PBS (100 μL/tube) for 30 min. During this time, the pellet was briefly agitated by hand to aid resuspension. Afterwards, the EV isolate was diluted six-fold in PBS and stored at 4 °C for a maximum of 48 h before use as “fresh EV isolate”.

### 2.3. EV Characterization

#### 2.3.1. Transmission Electron Microscopy

Ultrastructure imaging was performed using a JEM-1011 TEM (JEOL USA Inc., Peabody, MA, USA). Briefly, 5 μL of fresh EV isolate was applied to a 3 mm carbon coated grid and excess liquid was wicked away using filter paper. A 1% uranyl acetate solution (5 μL) was applied to the grid to stain lipids and increase the contrast of the EV membrane relative to the grid. The uranyl acetate solution was wicked away using filter paper. The carbon grids were imaged using the TEM at between 60 and 200× magnification.

#### 2.3.2. Dynamic Light Scattering

Dynamic light scattering (DLS) was performed using a Nano-ZS90 Zetasizer (Malvern Panalytical, Westborough, MA, USA). Briefly, 80 μL of fresh EV isolate was added to an ultra-micro 8.5 mm cuvette (Brandtech Scientific #759200, Essex, CT, USA). The cuvette was inserted into the Zetasizer and allowed to equilibrate to 25 °C for 60 s prior to particle measurement at an automatically determined attenuation and duration. EVs were presumed to have the same refractive index and absorption as protein suspended in water. Data were processed using the protein analysis model included with the Zetasizer software (v7.13). This model allows for a clear identification of adjacent peaks and interpretation of the measurements.

#### 2.3.3. Total Protein Concentration

Total protein content of the fresh EV isolate was quantified using a micro bicinchoninic acid (BCA) protein assay (Thermo Scientific # 23235, Waltham, MA, USA). Briefly, 50 μL of fresh EV isolate was diluted to 500 μL in either PBS or sodium dodecyl sulfate (SDS, final concentration 2%). Samples were vortexed for 30 s. SDS and vortexing was employed to lyse intact EVs and encourage the release of encapsulated protein cargo. 150 μL of EVs suspended in either PBS or SDS were added to the wells of a 96 well plate in triplicate. Then, 150 μL of working solution was added to each well in order to initiate the colormetric reaction. Two standard curves were also processed in triplicate for BSA suspended in either PBS or SDS at concentrations ranging from 0 to 200 μg/mL. The plate was incubated at 37 °C for 2 h, cooled to room temperature, and protein content was quantified by reading absorbance at 562 nm using a microplate reader.

### 2.4. SMC Cell Culture Conditions

Human aortic SMCs were obtained from ATCC (ATCC #PCS-100-012). All cell culture was performed at 37 °C and 5% CO_2_, with supplemented SMC growth media (SBM, Cell Applications Inc. #311K-500, San Diego, CA, USA) changed every 48–72 h. The cells used in the experimental cultures were between passages 4 and 12.

### 2.5. Proliferation Assays

Wells of 48-well plates were coated with collagen (type I rat tail collagen, Gibco #A10483) dissolved in 0.02 M acetic acid. Briefly, 500 μL of 50 μg/mL collagen solution was added to each well and placed at room temperature in sterile conditions for one hour. Wells were then washed 3 times in PBS and SMCs were plated at concentrations of 4000 cells per well respectively in 1 mL of NT media. After 16 h, the NT media was removed, and wells were washed with PBS. A baseline reading of cellular activity was obtained by adding 300 μL of unsupplemented basal SMC growth media (BM) and 30 μL of Alamar Blue (Invitrogen #DAL1100, Waltham, MA, USA) to each well for a final concentration of 11:1 and incubating the plate at 37 °C and 5% CO_2_ for 4 h. Then, 100 μL of solution was removed from each well and transferred to a 96-well plate where the absorbance of the product and reactant were read at 570 and 600 nm, respectively, using a plate reader. Readings are presented as 570/600 nm. Each well was washed with PBS and treatments comprised of 150 μL BM and 150 μL treatment (0, 50, or 150 μL EV isolate + PBS to reach 150 μL total or SBM) were applied to each well in triplicate. Following incubation for 24 h, treatments were removed, and each well was washed with PBS. Cell activity was measured using Alamar Blue as outlined above and cell activity after 24 h of treatment is normalized to baseline cell activity in order to account for well-to-well variance. Cell proliferation for each treatment is presented relative to the BM treatment group.

### 2.6. In Vitro Wound Closure Assays

SMCs were plated in 24 well plates at 50,000 cells per well in 2 mL of SBM media. After 16 h, 1 mL pipette tips were used to introduce a scratch in each well. SBM media was removed, and treatments comprised of 350 μL BM and 150 μL treatment (as defined in Section 2.5) were applied to wells in triplicate. The plate was transferred to a closed stage-top incubator (Tokai Hit Co., Bala Cynwyd, PA, USA) atop the motorized stage of an inverted Nikon TiE fluorescent microscope (Nikon, Inc., Melville, NY, USA) equipped with a 10×, 0.5 NA plan apochromatic lens (Nikon, Inc., Melville, NY, USA) and maintained at 37 °C and 5% CO_2_ for 24 h. Wells were imaged every hour for 48 h. Wound area was measured using ImageJ and wound closure is presented as the final would area relative to the initial wound area.

### 2.7. 3D SMC-Fibrin Gel Construct Fabrication

SMC-seeded fibrin gel constructs were formed as previously described [15] in “disc” and “strand” configurations using 3.7 mg/mL bovine fibrinogen type I (Sigma Aldrich #F8630, St. Louis, MI, USA), 0.21 U/mL bovine thrombin (Sigma Aldrich #605157, St. Louis, MI, USA), and 5 × 10^5^ SMCs/mL. Constructs were allowed to polymerize for one hour before the addition of SBM for 24 h prior to initial treatment. Treatment changes were made every 48–72 h. Specified treatments (see “Media treatment conditions” section) were used to culture 3D SMC constructs until harvest at 30-days post-fabrication. Aminocaproic acid (ACA) (Sigma Aldrich #07260, St. Louis, MI, USA), a lysine-mimicking fibrinolysis inhibitor, was added to all culture media at 12 mM (including the initial “pre-treatment” 24 h) to inhibit cell-driven degradation of the fibrin gel constructs.

### 2.8. Media Treatment Conditions for Fibrin Gel Constructs

All media used for fibrin gel construct treatment was composed of a 1:1 mix of BM and the desired treatment media. The negative control for all experiments was a 1:1 mix of BM and nCM, as described in Section 2.1 the latter was the non-conditioned culture media for ASCs. ASC conditioned media (CM) or EV-depleted conditioned media (dCM) treatment was prepared by freshly thawing CM or dCM, respectively, and mixing at a 1:1 ratio with BM. EV1× and EV3× treatments were prepared by mixing 3 μL and 9 μL of fresh EV isolate per mL of nCM, respectively, then mixing 1:1 with BM. These volumes of EV isolate were used as during one isolation; 600 μL of EV isolate is obtained from ~200 mL of CM, which amounts to 3 μL per mL of CM. Therefore, the EV1× treatment used in this study represents the concentration of EV isolate that should be present in the equivalent volume of CM and EV3× represents three times that concentration.

### 2.9. qPCR Analysis

Following sonication of frozen disc constructs, RNA collection (illustra RNAspin Mini Kit, GE Healthcare Life Sciences #25050070, Chicago, IL, USA) and RNA concentration quantification (Take3 BioTek, Winooski, VT, USA) were performed. After pre-heating template (65 °C, 5 min), synthesis of first-strand cDNA used random hexamer primers and the SuperScript IV First-Strand Synthesis System (Invitrogen #18091050, Waltham, MA, USA) (23 °C/10 min, 55 °C/10 min, 80 °C/10 min). RT-qPCR was performed using KiCqStart SYBR Green ReadyMix with ROX (Sigma-Aldrich #KCQS02) and previously published primers [15]. Post-amplification melt curves validated proper amplification.

### 2.10. Ninhydrin (Insoluble Elastin) and Hydroxyproline (Collagen) Assays

As established previously [15,23], base hydrolysis and subsequent centrifugation (3000× *g*) were used to separate insoluble elastin protein from soluble non-elastin protein (0.1M NaOH, 1 h at 98 °C). Acid hydrolysis (6N HCl, 24 h at 110 °C) and assay quantification on both soluble and insoluble fractions (ninhydrin-based for elastin, hydroxyproline-based for collagen) was used to quantify ECM content within each 3D construct. Fibrin gel constructs were frozen at −80 °C prior to ninhydrin/hydroxyproline analysis and thawed immediately before base hydrolysis.

### 2.11. Tensile Testing of Soft Substrate Fibrin Gel Constructs

Strand constructs were harvested, without fixation, by cutting the nylon tabs within the Linear TissueTrain plates, keeping each gel intact. Pure tensile testing was performed as the mean width to length ratio of the samples was 0.216 ± 0.074 (range: 0.026–0.409) [24]. The thickness, width and gauge length of the samples were measured using photos obtained prior to clamping, and again once clamped, to visualize sample dimensions in the presence of a calibrated ruler. The image processing software ImageJ (FIJI, public domain) was utilized to measure sample dimensions from these images. 

A uniaxial tensile testing device (Instron, #5543A, Norwood, MA, USA) was used to determine the tensile mechanical properties of the strands. The dried nylon tabs of each sample were secured in compression-based pneumatic clamps lined with sandpaper [15,25,26]. To eliminate slack, each sample was stretched to a pre-load of 0.01 N. A constant 0.1 mm/second crosshead extension speed was used until failure to characterize the mechanical behavior of the samples under quasi-static loading [27]. Force and displacement values were recorded throughout the test and converted to stress-stretch ratio plots [28] using the following equations:

Stretch ratio λ=LLo ; Stress σ=FAo

These measurements were calculated from the sample gauge length in the loaded (*L*) and unloaded configuration (*Lo*) along with the force (*F*) and original cross-sectional area (*Ao*) recorded during each mechanical test. The *Ao* of the gels was presumed to be an oval. Low and high moduli are defined as the slope of the linear portion of the mechanical response curve in the low and high stretch regions respectively. The transition between the low and high stretch regions is defined as the point of the stress–stretch ratio curve with the maximum normal distance from the global secant, which is the line spanning from the origin to the end of the curve [29,30]. This amounts to dividing the curves into three equal parts and treating the initial and final thirds of the curve as the low and high stretch regions respectively.

### 2.12. Statistical Analysis

Means comparisons were conducted using individual *t*-tests or one-way ANOVA with Tukey post-hoc tests, as appropriate. Significance threshold of α = 0.05 was set for all presented data, with experimental sample size of tested constructs available on each figure. Prism Graphpad was used for all statistical analysis. All displayed data values written after “±” are standard deviation values.

## 3. Results

### 3.1. EVs Secreted from ASCs Are Characteristic of Exosomes and Microvesicles

**Figure 1:**
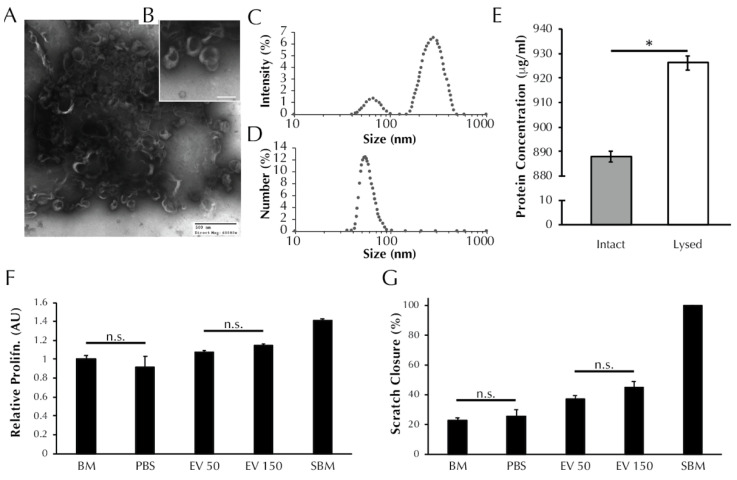
EV isolates are characterized by the presence of exosome-like particles and have a positive effect on both SMC proliferation and migration. (**A**) TEM images of ASC secreted EVs visible at 60× and (**B**) 150× magnification resolutions. Black shadowing is free protein present within the EV isolate resuspension. (**C**) Size distribution of particles within the EV isolate as determined using dynamic light scattering in terms of intensity and (**D**) number of readings. (**E**) Total protein content of the EV isolate before and after lysis (* = *p* < 0.0001 by *t*-test). (**F**) SMC proliferation and (**G**) migration after treatment with unsupplemented basal media (BM), PBS vehicle control, or increasing concentrations of EVs (50 or 150 μL). Aside from pairs marked with “n.s.” all pairs were significantly different by one-way ANOVA, *p* < 0.05.

TEM analysis revealed nanoparticles in the EV isolate that exhibit the cup-like morphology typical of EVs [31], Figure 1A,B. DLS analysis revealed the presence of two populations of particles within the EV isolate that exhibit different diameters (Figure 1C), with max peaks at 66 nm and 283 nm, which are indicative of exosomes and microvesicles, respectively [31]. The number-diameter distribution of particles within the EV isolate as a percentage of the total measurements shows that the two peaks are reduced to a single peak at 54.08 nm. The single peak in Figure 1D indicates that although the EV isolate contains two size populations of particles, the exosome sized particles occur more frequently by number. Total protein analysis (Figure 1E) revealed that the protein concentration of the lysed EV isolate is greater than the intact EV isolate (887.9 ± 2.2 vs. 926.3 ± 2.9 μg/mL, *p* < 0.0001). The BCA data support the preceding TEM and DLS evidence to suggest that EVs are present within the EV isolate and that the EVs are encapsulating protein.

### 3.2. ASC-Derived EVs Promote SMC Proliferation

As presented in Figure 1F, treatment with 50 μL of EV isolate induced no change in SMC proliferation relative to BM control (1.08 ± 0.02 vs. 1.00 ± 0.04 AU, *p* > 0.05). Treatment with 150 μL of EV isolate induced an increase in proliferation relative to BM (1.14 ± 0.02 vs. 1.00 ± 0.04 AU, *p* > 0.01). Both EV treatment groups exhibited proliferation levels lower than supplemented basal media (SBM) positive control (1.41 ± 0.02 AU, *p* < 0.0001), and both EV treatment groups were increased compared to PBS vehicle control (0.92 ± 0.11 AU, *p* < 0.0001).

### 3.3. ASC-Derived EVs Promote SMC Migration in an In Vitro Scratch Wound Assay

As presented in Figure 1G, SMC migration following treatment with 50 μL (37.0 ± 2.5% wound closure) and 150 μL (45.1 ± 3.8%) of EV isolate were increased relative to both the negative control BM (22.8 ± 1.7%, *p* < 0.05) and the vehicle control PBS (25.3 ± 4.5%, *p* < 0.05). Migration in the presence of the positive control, SBM, saw full wound closure in all replicate wells (100 ± 0%) and was higher than both EV treatment groups and both negative controls (*p* < 0.001).

### 3.4. ASC-Derived EVs Had a Minimal Effect on Elastic Fiber Gene Transcription within SMC Disc Constructs after 30 Days of Culture

**Figure 2:**
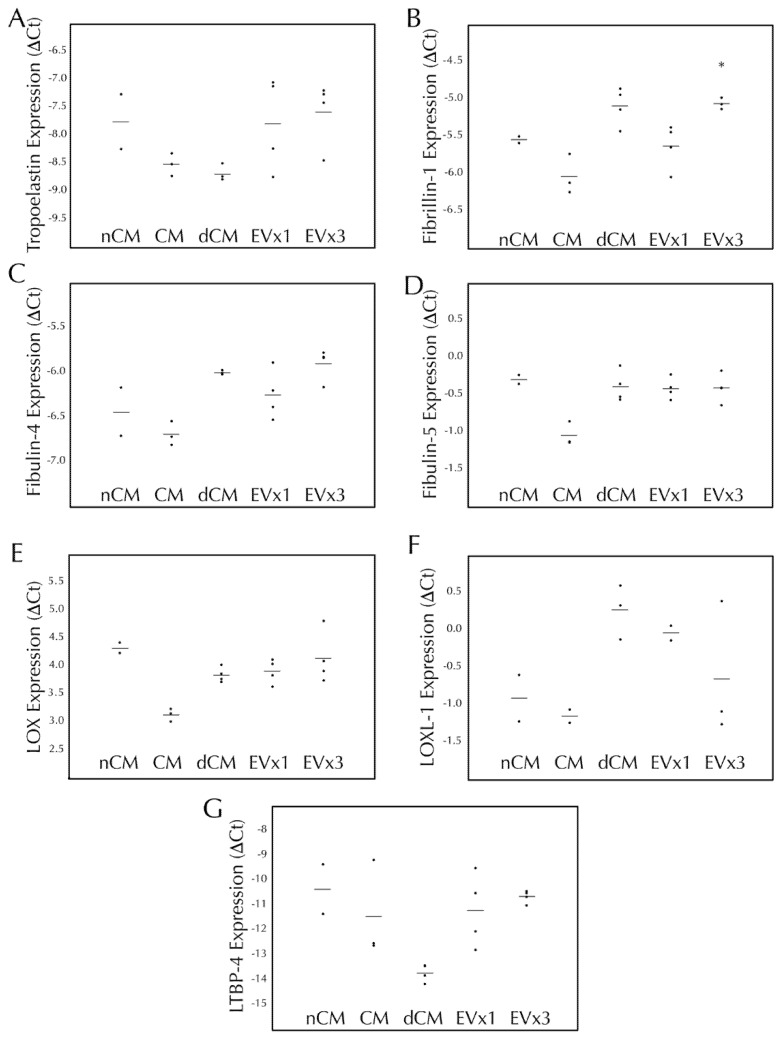
3×EV treatment of discs lead to increased transcription of fibrillin-1 after 30 days of culture. Fibrin discs were cultured for 30 days using treatments replenished every 48–72 h, subjected to RNA extraction, and analyzed by RT-qPCR for expression of human (**A**) tropoelastin, (**B**) fi-brillin-1, (**C**) fibulin-4, (**D**) fibulin-5, (**E**) lysyl oxidase (LOX), (**F**) lysyl oxidase-like 1 (LOXL-1), and (**G**) latent TGF-β binding protein-4 (LTBP-4). Treatment groups were non-conditioned media (nCM), ASC conditioned media (CM), CM depleted of EVs (dCM), 1× extracellular vesicle treatment (EV×1), or 3×EV treatment (EV×3). Gene expression was expressed as ΔCt relative to the housekeeping gene GAPDH, where higher values relative to nCM indicate higher expression levels. For all groups, the individual data points are plotted as dots, with a line indicating the average value. * = *p* < 0.05 relative to nCM.

Elastic fiber formation and maturation involves multiple genes in addition to the gene for tropoelastin. Therefore, transcription of tropoelastin, fibrillin-1, fibulin-4, fibulin-5, LOX, LOXL-1, and LTBP-4 were monitored following 30 days of EV treatment. As presented in Figure 2, SMC transcription of tropoelastin, fibulin-4, fibulin-5, LOX, LOXL-1, and LTBP-4 were all unchanged after 30 days of EV treatment relative to the negative control, nCM. CM led to a decrease in fibulin-5 expression while both CM and dCM led to an increase in LOX expression. Upregulation of fibrillin-1 was observed with 3×, but not 1×, EV treatment (an increase of 39.6 ± 6.2%, *p* < 0.05) as well as dCM, Figure 2B.

### 3.5. ASC-Derived EVs Promote Insoluble Elastin and Collagen Deposition within SMC Disc Constructs after 30 Days of Culture

**Figure 3:**
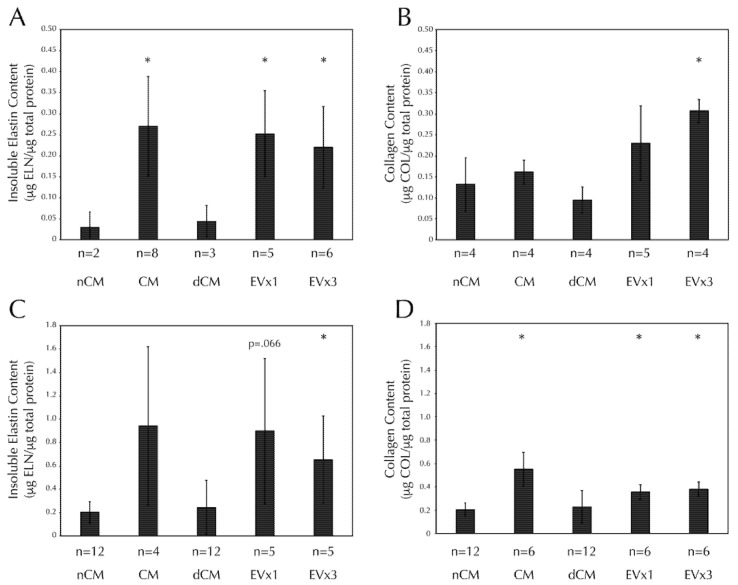
SMC insoluble elastin and collagen deposition in both discs and strands are increased after 30 days of CM or EV treatment, but not dCM. All fibrin constructs were cultured as in Figure 2. (**A**) Insoluble elastin deposition by Scheme 30. days of stimulation with CM, EV×1, and EV×3 relative to the negative control, nCM. (**B**) Total collagen deposition by SMCs in fibrin discs was increased after 30 days of stimulation with 3×EV relative to nCM. (**C**) Insoluble elastin deposition by SMCs in fibrin strands was increased after 30 days of stimulation with 3×EV relative to nCM. (**D**) Total collagen deposition by SMCs in fibrin strands was increased after 30 days of stimulation with CM, EV×1, and EV×3 relative to nCM. Sample size is indicated for each group. * = *p* < 0.05 relative to nCM.

Insoluble elastin deposition was increased in discs treated with CM, 1×EV, and 3×EV relative to the negative control, nCM, Figure 3A. Collagen deposition was also increased in discs treated with 3×EV relative to nCM, Figure 3B. Notably, EV-depleted CM (dCM) was unable to promote ECM deposition similarly in either case.

### 3.6. ASC-Derived EVs Promote Insoluble Elastin and Collagen Deposition within SMC Strand Constructs after 30 Days of Culture

Insoluble elastin deposition was increased in strands treated with 3×EV, relative to nCM, Figure 3C. Trends towards significance were seen in both CM (*p* = 0.12) and 1×EV (*p* = 0.066). Collagen deposition was increased in strands treated with CM, 1×EV, and 3×EV relative to nCM, Figure 3D. Again, the depletion of EVs from the CM prevented the dCM from promoting the deposition of either ECM component.

### 3.7. ASC-Derived EVs Did Not Affect the Modulus in the Low or High Stretch Regions of Strands after 30 Days of Culture

**Figure 4:**
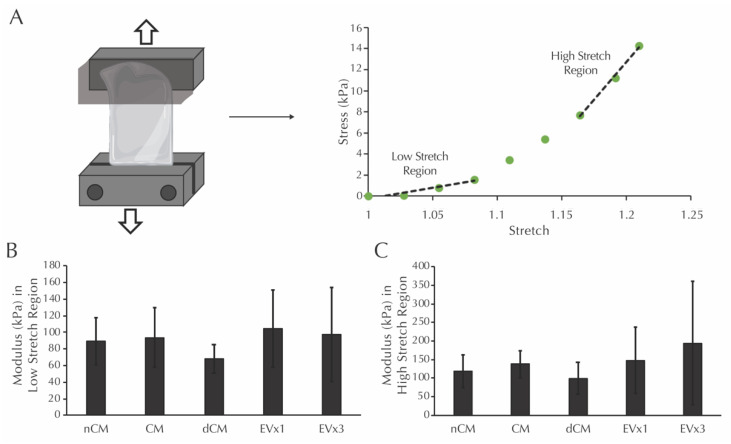
Moduli in the low and high stretch regions for fibrin strands are not increased after 30 days of EV-containing treatment groups. (**A**) Schematic of the mechanical testing procedure for fibrin strands and the definition of low and high stretch regions. (**B**) Modulus of the system in the low stretch region following culture with the treatments described in Figure 2. (**C**) Modulus of the system in the high stretch region following culture with the same treatments. No significant differences were detected using one-way ANOVA.

Prior to mechanical testing, the dimensions of each strand were measured. Average values for thickness, width, and gauge length were calculated from these measurements: thickness (1.13 ± 0.25 mm NCM, 1.15 ± 0.20 mm dCM, 1.25 ± 0.25 mm CM, 1.10 ± 0.48 mm 1×EV, 1.16 ± 0.18 mm 3×EV), width (2.14 ± 0.31 mm NCM, 1.93 ± 0.46 mm dCM, 2.39 ± 0.37 mm CM, 2.00 ± 0.42 mm 1×EV, 2.01 ± 0.49 mm 3×EV), and gauge length (10.74 ± 2.44 mm NCM, 10.31 ± 2.90 mm dCM, 11.06 ± 1.97 mm CM, 7.86 ± 1.81 mm 1×EV, 10.40 ± 2.64 mm 3×EV). Moduli in the low and high stretch regions, defined as the slope of the given sample’s mechanical response curve within the low stretch range (initial third of the curve) and high stretch range (final third of the curve), were then analyzed, as shown in Figure 4A. After 30 days, the moduli in the low stretch region were equivalent in all groups, including the nCM negative control, Figure 4B. Moduli in the high stretch region were similarly equivalent in all groups, Figure 4C.

## 4. Discussion

This study utilizes a 3D culture system to demonstrate for the first time that EVs derived from ASC conditioned media with characteristics of exosomes and microvesicles (Figure 1) can be used to induce production of two important vascular ECM components by SMCs, specifically elastin and fibrillar collagen. While gene expression analysis after 30 days only reflected a prolonged elevation of fibrillin-1 transcription by media supplementation with 3×EV treatment (Figure 2), the elastin and collagen ECM contents of both discs and strands was elevated by supplementation of 1× or 3×EVs, similar to what has been previously reported by our group for CM [15] (Figure 3). The uniaxial elastic moduli of strand constructs, however, were not affected by EV treatment (Figure 4). Our findings highlight the potential of employing ASC derived EVs as a tool for inducing the deposition of structural ECM proteins.

The production of elastin and collagen matrix by SMCs is a highly desirable target for therapies aimed at arresting aneurysm growth and fabricating de novo vascular tissue in situ. Although effective non-surgical options for aneurysm repair have yet to be developed [4,5], our group has investigated cell-based therapies to restore elastin in murine models of elastase induced aneurysm. We previously demonstrated that administering 100,000 ASCs periadventitially to a murine aneurysm model can preserve elastin fiber integrity [32]. In an attempt to move towards a cell-free therapeutic, we have also shown an increase in elastin and collagen deposition by SMC following treatment with full ASC secretome using the same 3D gel culture system used in this study [15]. Our present study advances the field by establishing for the first time that the EV fraction of ASC conditioned media can be isolated and utilized as a defined therapeutic tool to induce ECM structural protein deposition. One point of difference to our previous study is that the FBS we use here to supplement the culture medium was depleted of EVs in order to better isolate the effects exhibited by ASC-EVs. EV-depletion of FBS may have attenuated the deposition of elastin and collagen in the ASC CM group in this study compared to our previous study [15]. However, the aim of developing cell-free therapeutics should be to completely remove animal-based products and employ highly defined cell culture media that can be used to develop a standardized product with reliable and reproducible therapeutic effects. Future studies should therefore attempt to remove FBS entirely from the culture procedure.

To address the shortcomings of autologous and synthetic materials available for bypassing grafting [7,8,9,10], many studies have attempted to develop tissue engineered vascular grafting systems that combine biodegradable scaffolds with stem cells that remodel into de novo vascular tissue in situ, as reviewed in [12]. EVs may represent a more translatable cell-free tool to combine with biodegradable scaffolds intended for vascular grafting applications as we demonstrate here that they are capable of encouraging deposition of ECM structural proteins and therefore likely to encourage positive remodeling of an implanted graft. Indeed, our group has already shown that incorporating EVs into vascular grafts results in increased elastin and collagen production compared to blank scaffolds [33]. The matrix synthesizing effects exhibited by EVs in this study are therefore readily transferable to the production of de novo vascular graft tissue in situ. Furthermore, this study illustrates the transcriptional pathways by which the in vivo effects are achieved.

There are several limitations to the present study. The first is regarding the isolation method used to obtain EVs from ASC conditioned media. The isolation of EVs can be performed using centrifugation, immune capture methods, microfluidic platforms, size- or density- gradient manipulation sorting and filtration methods, or through precipitation kits that alter EV solubility. Isolation via ultracentrifugation, as done in this study, improves overall EV yield in comparison to filtration-based and immune-based methods, but is susceptible to protein contamination [34]. Indeed, the dark shadowing seen in Figure 1A,B indicates the presence of free protein that persisted past ultracentrifugation. Protein assay levels seen in Figure 1E also confirm the presence of free protein, with considerable protein content present before EV lysing was performed to release EV-encapsulated protein. However, as there is no ‘gold-standard’ method for isolating EVs, the method chosen should reflect the intended purpose of the EVs [35]. We therefore employ one ultracentrifugation step because it allows for a large volume of conditioned media to be processed in a relatively short timeframe. Repeat ultracentrifugation, intended to increase EV isolate purity, was avoided as this method has been shown to decrease EV yield due to incomplete sedimentation and aggregation of EVs [36]. Despite this reasoning, future studies wishing to refine our EV-based tool could attempt alternative methods that allow for the isolation of a purer EV fraction. Second, while we did not see an effect on ECM transcription at the 30-day timepoint, it is possible that the cells have adapted transcriptionally to the stimulatory effect of the EVs and CM. Ultimately, the ECM content is the most critical element of this study, so this limitation may be minor. However, dissecting the exact order of transcriptional events for the multiple matricellular proteins responsible for elastic fiber assembly [11,15] will be a topic of future study by our team. Third, the manner of delivering our EV-based therapeutic in this study is inherently unfeasible in a clinical setting where the aim is to arrest aneurysm growth or stimulate the growth of de novo vascular tissue. The development of a therapeutic that induces ECM deposition necessitates a sophisticated delivery system due to the speed of clearance in vivo. Future studies must investigate an effective way of delivering EVs to the intended site in a one off, or limited series of doses. However, the stability of EVs and their resistance to extended storage periods makes them an ideal candidate for an off-the-shelf treatment [12] that could be easily administered once an effective delivery system is developed. Fourth, there is an apparent disconnect between deposited insoluble elastin and a downstream effect on elastic modulus. In fact, this is consistent with our previous observation that increased elastin deposition in ASC-CM treated strands does not elicit a significant change in modulus (in the low stretch region) compared to controls [15]. Elastin, a linearly elastic material, influences the mechanical response of vascular tissue in the low stretch region; whereby elastin fibers bear the applied load, prior to decrimping and straightening of coiled collagen fibers which then assume the load [37]. The findings of this study suggest that the elastin present in CM and EV treated strands exhibits a statistically similar mechanical function to the control groups despite increased deposition in the former groups. It is possible that insufficient elastin has been deposited within the constructs to elicit a change in functional mechanical response. Furthermore, this study reports on the elastic (instantaneous) mechanical response of the strands. However, elastin also influences the viscous (time-dependent) mechanical response of biological materials [38]. It is therefore possible that despite displaying similar moduli in the low-stretch region, the treated groups could display more physiologically relevant viscous mechanical properties (such as increased creep resistance) relative to controls. Finally, although we observe a potent matrix synthesizing effect when administering EVs to SMCs in this study, we do not characterize the cargo of the EVs that may be responsible for causing this effect. However, previous work has demonstrated that ASC derived EVs are enriched for miR-183, miR-378, miR-140, and miR-222; 255; mRNAs including TRPS1, ELK4, KLF7 and NRIP1; and 277 proteins including glycoproteins and extracellular matrix proteins, and TGF-β signaling [19]. It has been shown that these EVs are specifically enriched in cargo for extracellular matrix remodeling such as MMP9 and TGFβ-1 [18,20]. Further characterization of the ASC secretome and EV cargo may elucidate the mechanism for elastogenesis induction.

This study demonstrates the first steps towards developing an EV-based tool for inducing the deposition of structural ECM proteins that could potentially be utilized in arresting aneurysm growth and fabricating de novo vascular tissue in situ. We feel that the results of this study warrant further investigation regarding delivery and transitioning towards in vivo studies that attempt to validate the promising in vitro results presented herein.

## 5. Conclusions

In this work, we hypothesized that EVs derived from ASCs would have a potent ECM protein synthesis effect on vascular SMCs. Using a 3D fibrin-gel culture system, we demonstrated that treatment with EVs increases the deposition of insoluble elastin and collagen in two different fibrin construct geometries, discs and strands, but does not ultimately lead to alterations in the elastic modulus of the uniaxially tested strands. The findings of this study therefore support our hypothesis that EVs promote vascular ECM synthesis and could potentially be of use in vascular tissue engineering and regenerative medicine solutions for vascular disease. Figure 4A was partially created using Biorender.

## Data Availability

The data presented in this study are available on request from the corresponding author.

## Abbreviations

List of abbreviations used in this manuscript

Abbreviation	Definition
AA	Aortic aneurysm
ACA	ε-amino caproic acid
ASC	Adipose-derived stromal cell
BCA	Bicinchoninic acid
BM	Basal SMC media
BSA	Bovine serum albumin
CM	Conditioned ASC culture media
CVD	Cardiovascular disease
dCM	CM depleted of extracellular vesicles
DLS	Dynamic light scattering
ECM	Extracellular matrix
EV	Extracellular vesicle
FBS	Fetal bovine serum
nCM	Non-conditioned ASC culture media
PBS	Phosphate buffered saline
SBM	Supplemented SMC growth media
SDS	Sodium dodecyl sulfate
SMC	Aortic smooth muscle cell
TEM	Transmission electron microscopy

## References

[1] Virani S.S., Alonso A., Benjamin E.J., Bittencourt M.S., Callaway C.W., Carson A.P., Chamberlain A.M., Chang A.R., Cheng S., Delling F.N. (2020). American Heart Association Council on E, Prevention Statistics C, Stroke Statistics S. Heart Disease and Stroke Statistics-2020 Update: A Report from the American Heart Association. Circulation.

[2] Goodney P.P., Beck A.W., Nagle J., Welch H.G., Zwolak R.M. (2009). National trends in lower extremity bypass surgery, endovascular interventions, and major amputations. J. Vasc. Surg..

[3] Alexander J.H., Smith P.K. (2016). Coronary-Artery Bypass Grafting. N. Engl. J. Med..

[4] Chun A.S., Elefteriades J.A., Mukherjee S.K. (2013). Do beta-Blockers Really Work for Prevention of Aortic Aneurysms?: Time for Reassessment. Aorta (Stamford).

[5] Baxter B.T., Terrin M.C., Dalman R.L. (2008). Medical management of small abdominal aortic aneurysms. Circulation.

[6] Nicholls S.C., Gardner J.B., Meissner M.H., Johansen H.K. (1998). Rupture in small abdominal aortic aneurysms. J. Vasc. Surg..

[7] Isenberg B.C., Williams C., Tranquillo R.T. (2006). Small-diameter artificial arteries engineered in vitro. Circ. Res..

[8] Harskamp R.E., Alexander J.H., Ferguson T.B., Hager R., Mack M.J., Englum B., Wojdyla D., Schulte P.J., Kouchoukos N.T., de Winter R.J. (2016). Frequency and Predictors of Internal Mammary Artery Graft Failure and Subsequent Clinical Outcomes: Insights From the Project of Ex-vivo Vein Graft Engineering via Transfection (PREVENT) IV Trial. Circulation.

[9] Desai M., Seifalian A.M., Hamilton G. (2011). Role of prosthetic conduits in coronary artery bypass grafting. Eur. J. Cardiothorac. Surg..

[10] Klinkert P., Post P.N., Breslau P.J., van Bockel J.H. (2004). Saphenous vein versus PTFE for above-knee femoropopliteal bypass. A review of the literature. Eur. J. Vasc. Endovasc. Surg..

[11] Ramaswamy A.K., Vorp D.A., Weinbaum J.S. (2019). Functional Vascular Tissue Engineering Inspired by Matricellular Proteins. Front. Cardiovasc. Med..

[12] Cunnane E.M., Weinbaum J.S., O’Brien F.J., Vorp D.A. (2018). Future Perspectives on the Role of Stem Cells and Extracellular Vesicles in Vascular Tissue Regeneration. Front. Cardiovasc. Med..

[13] Saitow C.B., Wise S.G., Weiss A.S., Castellot J.J., Kaplan D.L. (2013). Elastin biology and tissue engineering with adult cells. Biomol. Concepts.

[14] Kelleher C.M., McLean S.E., Mecham R.P. (2004). Vascular extracellular matrix and aortic development. Curr. Top. Dev. Biol..

[15] Ramaswamy A.K., Sides R.E., Cunnane E.M., Lorentz K.L., Reines L.M., Vorp D.A., Weinbaum J.S. (2019). Adipose-derived stromal cell secreted factors induce the elastogenesis cascade within 3D aortic smooth muscle cell constructs. Matrix Biol. Plus..

[16] Sharma A.K., Salmon M.D., Lu G., Su G., Pope N.H., Smith J.R., Weiss M.L., Upchurch G.R. (2016). Mesenchymal Stem Cells Attenuate NADPH Oxidase-Dependent High Mobility Group Box 1 Production and Inhibit Abdominal Aortic Aneurysms. Arterioscler. Thromb. Vasc. Biol..

[17] Spinosa M., Lu G., Su G., Bontha S.V., Gehrau R., Salmon M.D., Smith J.R., Weiss M.L., Mas V.R., Upchurch G.R. (2018). Human mesenchymal stromal cell-derived extracellular vesicles attenuate aortic aneurysm formation and macrophage activation via microRNA-147. FASEB J..

[18] Eirin A., Riester S.M., Zhu X.Y., Tang H., Evans J.M., O’Brien D., van Wijnen A.J., Lerman L.O. (2014). MicroRNA and mRNA cargo of extracellular vesicles from porcine adipose tissue-derived mesenchymal stem cells. Gene.

[19] Eirin A., Zhu X.Y., Puranik A.S., Woollard J.R., Tang H., Dasari S., Lerman A., van Wijnen A.J., Lerman L.O. (2017). Integrated transcriptomic and proteomic analysis of the molecular cargo of extracellular vesicles derived from porcine adipose tissue-derived mesenchymal stem cells. PLoS ONE.

[20] Eirin A., Zhu X.Y., Puranik A.S., Woollard J.R., Tang H., Dasari S., Lerman A., van Wijnen A.J., Lerman L.O. (2016). Comparative proteomic analysis of extracellular vesicles isolated from porcine adipose tissue-derived mesenchymal stem/stromal cells. Sci. Rep..

[21] Haskett D.G., Saleh K.S., Lorentz K.L., Josowitz A.D., Luketich S.K., Weinbaum J.S., Kokai L.E., D’Amore A., Marra K.G., Rubin J.P. (2018). An exploratory study on the preparation and evaluation of a "same-day" adipose stem cell-based tissue-engineered vascular graft. J. Thorac. Cardiovasc. Surg..

[22] Krawiec J.T., Liao H.T., Kwan L.L., D’Amore A., Weinbaum J.S., Rubin J.P., Wagner W.R., Vorp D.A. (2017). Evaluation of the stromal vascular fraction of adipose tissue as the basis for a stem cell-based tissue-engineered vascular graft. J. Vasc. Surg..

[23] Ahmann K.A., Weinbaum J.S., Johnson S.L., Tranquillo R.T. (2010). Fibrin degradation enhances vascular smooth muscle cell proliferation and matrix deposition in fibrin-based tissue constructs fabricated in vitro. Tissue Eng. Part A.

[24] Mulvihill J.J., Walsh M.T. (2013). On the mechanical behaviour of carotid artery plaques: The influence of curve-fitting experimental data on numerical model results. Biomech. Model. Mechanobiol..

[25] Blose K.J., Pichamuthu J.E., Weinbaum J.S., Vorp D.A. (2016). Design and Validation of a Vacuum Assisted Anchorage for the Uniaxial Tensile Testing of Soft Materials. Soft Mater..

[26] Tsamis A., Phillippi J.A., Koch R.G., Pasta S., D’Amore A., Watkins S.C., Wagner W.R., Gleason T.G., Vorp D.A. (2013). Fiber micro-architecture in the longitudinal-radial and circumferential-radial planes of ascending thoracic aortic aneurysm media. J. Biomech..

[27] Holzapfel G.A., Sommer G., Regitnig P. (2004). Anisotropic mechanical properties of tissue components in human atherosclerotic plaques. J. Biomech. Eng..

[28] Walsh M.T., Cunnane E.M., Mulvihill J.J., Akyildiz A.C., Gijsen F.J., Holzapfel G.A. (2014). Uniaxial tensile testing approaches for characterisation of atherosclerotic plaques. J. Biomech..

[29] Holzapfel G.A. (2006). Determination of material models for arterial walls from uniaxial extension tests and histological structure. J. Theor. Biol..

[30] Cunnane E.M., Mulvihill J.J.E., Barrett H.E., Hennessy M.M., Kavanagh E.G., Walsh M.T. (2016). Mechanical properties and composition of carotid and femoral atherosclerotic plaques: A comparative study. J. Biomech..

[31] Thery C., Ostrowski M., Segura E. (2009). Membrane vesicles as conveyors of immune responses. Nat. Rev. Immunol..

[32] Blose K.J., Ennis T.L., Arif B., Weinbaum J.S., Curci J.A., Vorp D.A. (2014). Periadventitial adipose-derived stem cell treatment halts elastase-induced abdominal aortic aneurysm progression. Regen. Med..

[33] Cunnane E.M., Lorentz K.L., Ramaswamy A.K., Gupta P., Mandal B.B., O’Brien F.J., Weinbaum J.S., Vorp D.A. (2020). Extracellular Vesicles Enhance the Remodeling of Cell-Free Silk Vascular Scaffolds in Rat Aortae. ACS Appl. Mater. Interfaces.

[34] Li P., Kaslan M., Lee S.H., Yao J., Gao Z. (2017). Progress in Exosome Isolation Techniques. Theranostics.

[35] Lotvall J., Hill A.F., Hochberg F., Buzas E.I., Di Vizio D., Gardiner C., Gho Y.S., Kurochkin I.V., Mathivanan S., Quesenberry P. (2014). Minimal experimental requirements for definition of extracellular vesicles and their functions: A position statement from the International Society for Extracellular Vesicles. J. Extracell. Vesicles.

[36] Webber J., Clayton A. (2013). How pure are your vesicles?. J. Extracell. Vesicles.

[37] Collins M.J., Eberth J.F., Wilson E., Humphrey J.D. (2012). Acute mechanical effects of elastase on the infrarenal mouse aorta: Implications for models of aneurysms. J. Biomech..

[38] Ryan A.J., O’Brien F.J. (2015). Insoluble elastin reduces collagen scaffold stiffness, improves viscoelastic properties, and induces a contractile phenotype in smooth muscle cells. Biomaterials.

